# Supplementary material for: Mechanisms governing the pioneering and redistribution capabilities of the non-classical pioneer PU.1
Source: Nat Commun. 2020 Jan 21;11:402. doi: 10.1038/s41467-019-13960-2 (PMC6972792; doi:10.1038/s41467-019-13960-2)
Supplement: Supplementary file 7 — Source data [file 41467_2019_13960_MOESM7_ESM.zip › Source_Data/FigureS2/FigureS2B_MotifScanOutput/KmeansCluster14/homerResults/motif5.similar.html]

motif5

## Information for motif5

G
T
C
A
A
G
T
C
A
G
T
C
T
C
G
A
A
T
G
C
T
C
G
A
C
T
G
A
C
G
A
T
C
A
G
T
A
G
T
C
A
G
T
C
A
G
T
C
  
Reverse Opposite:  

A
C
T
G
A
C
T
G
C
T
A
G
G
T
C
A
G
C
T
A
G
A
C
T
A
G
C
T
A
T
C
G
A
G
C
T
C
T
A
G
A
C
T
G
A
C
G
T
  

|  |  |
| --- | --- |
| p-value: | 1e-27 |
| log p-value: | -6.367e+01 |
| Information Content per bp: | 1.708 |
| Number of Target Sequences with motif | 55.0 |
| Percentage of Target Sequences with motif | 10.78% |
| Number of Background Sequences with motif | 692.7 |
| Percentage of Background Sequences with motif | 1.53% |
| Average Position of motif in Targets | 87.3 +/- 51.1bp |
| Average Position of motif in Background | 93.8 +/- 68.7bp |
| Strand Bias (log2 ratio + to - strand density) | 0.1 |
| Multiplicity (# of sites on avg that occur together) | 1.20 |
| Motif File: | file (matrix) reverse opposite |

### Similar de novo motifs found

|  |  |  |  |  |  |  |  |
| --- | --- | --- | --- | --- | --- | --- | --- |
| Rank | Match Score | Redundant Motif | P-value | log P-value | % of Targets | % of Background | Motif file |
| 1 | 0.898 | C T A G T C G A A G T C A G C T C G T A A G T C T G C A A G C T G C A T G A C T A G T C A G T C A G T C | 1e-23 | -53.701085 | 7.65% | 0.84% | motif file (matrix) |
| 2 | 0.851 | C T A G C T G A A G T C A G C T G T C A A G T C C T G A C A G T G A C T G C A T A G T C A G T C A G T C C T G A | 1e-20 | -48.337737 | 5.29% | 0.36% | motif file (matrix) |
| 3 | 0.855 | A G T C A C G T T C G A A G T C G T C A C T G A G C A T A G C T A G T C A G T C A G T C | 1e-14 | -32.304338 | 4.12% | 0.39% | motif file (matrix) |
